# Apples to Apples: Comparing PM_2.5_ Exposures and Birth Outcomes in Understudied Countries

**DOI:** 10.1289/ehp.122-A110

**Published:** 2014-04-01

**Authors:** Julia R. Barrett

**Affiliations:** Julia R. Barrett, MS, ELS, a Madison, WI–based science writer and editor, has written for *EHP* since 1996. She is a member of the National Association of Science Writers and the Board of Editors in the Life Sciences.

Mixed evidence suggests inhalation of fine particulate matter (PM_2.5_), a component of air pollution, may adversely affect birth outcomes, and research to elucidate the potential connection is ongoing.^1^ A new study in *EHP* provides further evidence for previously described associations between PM_2.5_ levels and low birth weight,^2^^,^^3^ but it goes a step farther by focusing on low- and middle-income countries, which have generally been excluded from previous analyses.^1^

**Figure:**
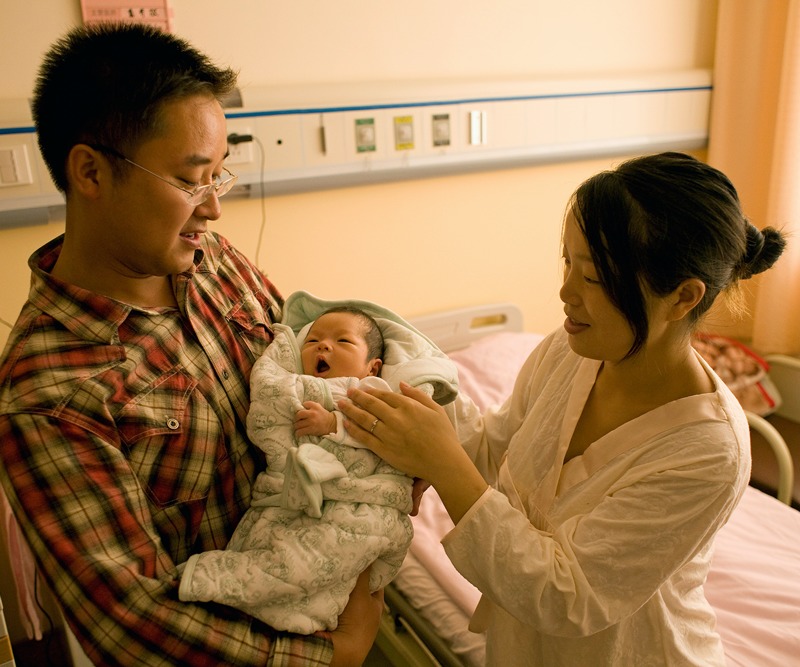
Only in China was increased estimated PM_2.5_ exposure associated with higher likelihood of both preterm birth and low birth weight. China had the highest estimated average PM_2.5_ exposures of the 22 countries studied. © Ryan Pyle/Corbis

In pregnant women, PM_2.5_ may provoke oxidative stress and inflammation, cause endocrine disruption, and impair oxygen transport across the placenta, all of which can potentially lead to low birth weight (defined as less than 2,500 g) and preterm birth (before the 37th week of pregnancy).^1^^,^^4^ Aside from the immediate consequences in infancy, low birth weight and preterm birth can affect health throughout childhood and in adulthood.^3^^,^^5^^,^^6^^,^^7^

The exclusion of low- and middle-income countries from earlier studies arose largely from a lack of dependable data. Health-related research on air pollution relies heavily on data gathered from air monitoring stations, which are most often placed in urban areas in developed countries.^8^^,^^9^ These stations provide precise data for air pollutant concentrations at particular locations. Exposure beyond these points can be extrapolated by adjusting for factors such as traffic and meteorological patterns, but only to a certain extent.^5^

The current study took a different approach, using 2001–2006 satellite data^8^ to estimate maternal PM_2.5_ exposures in the month prior to birth; where available, ground-monitoring data were used to confirm satellite-based estimates. The study included data on 192,900 live births in 22 countries in Africa, Latin America, and Asia, which were collected through the World Health Organization Global Survey on Maternal and Perinatal Health (WHOGS).^10^ Data included standard information on all births at participating health care facilities during 2- to 3-month periods between September 2004 and April 2008.

The statistical analysis used two models. The first provided a global estimate for all countries, while controlling for maternal factors (e.g., age, education, parity) and infant sex. The second additionally controlled for country-specific factors, such as income, urbanicity, availability of prenatal care, and per-capita health care expenditures as proxies for broader development.

Results from both models showed that higher levels of PM_2.5_ were associated with birth weight reduction but not preterm birth, with one exception: “In rapidly developing countries with high levels of air pollution, such as China, the highest levels of air pollution may be of concern for both preterm birth and low birth weight,” says lead author Nancy L. Fleischer, an assistant professor of epidemiology and biostatistics at the University of South Carolina.

A key strength of the study is that it used a standard data-collection method across multiple low- and middle-income countries. The study also had several limitations. “The use of satellite imaging data is a very clever idea,” says David A. Savitz, a professor of epidemiology and of obstetrics and gynecology at Brown University. “But there were real compromises that had to be made to do so—mainly that they could only examine spatial, not temporal variation; were not able to align exposure periods with the pregnancies precisely; and assigned the same exposure score within fifty kilometers of the clinic at which the women received care without being able to take into account where they lived,” he says.

The researchers discuss these limitations in their report, and also point out the lack of data on indoor air pollutants and the potential influence of unmeasured confounding factors. “Hopefully, this research will spur further air pollution studies in low- and middle-income countries that can collect better measurements of both local, detailed data on air pollution and birth outcomes,” says Fleischer.

Savitz agrees that further research with more reliable measurements is needed. “I can see two avenues for progress,” he says. “One is to identify the truly susceptible among whom exposure causes a substantial increase in risk, not the subtle effects reported in most studies. The other avenue would be more mechanistic studies that can tightly demonstrate the intermediate links between air pollution and pathways known to affect fetal growth.”
